# Refractory Esophageal Strictures: What To Do When Dilation Fails

**DOI:** 10.1007/s11938-014-0043-6

**Published:** 2015-02-04

**Authors:** Petra G. A. van Boeckel, Peter D. Siersema

**Affiliations:** Department of Gastroenterology and Hepatology, HP: F02.618, University Medical Center, Heidelberglaan 100, 3584 CX Utrecht, Netherlands

**Keywords:** Refractory benign esophageal stricture, Dysphagia, Dilation, Incisional therapy, Intralesional steroid injection, Stent placement, Self-expandable plastic stent, Self-expandable metal stent, Biodegradable stent placement, Esophagectomy, Self-bougienage

## Abstract

Benign esophageal strictures arise from a diversity of causes, for example esophagogastric reflux, esophageal resection, radiation therapy, ablative therapy, or the ingestion of a corrosive substance. Most strictures can be treated successfully with endoscopic dilation using bougies or balloons, with only a few complications. Nonetheless, approximately one third of patients develop recurrent symptoms after dilation within the first year. The majority of these patients are managed with repeat dilations, depending on their complexity. Dilation combined with intra lesional steroid injections can be considered for peptic strictures, while incisional therapy has been demonstrated to be effective for Schatzki rings and anastomotic strictures. When these therapeutic options do not resolve the stenosis, stent placement should be considered. Self bougienage can be proposed to a selected group of patients with a proximal stenosis. As a final step surgery is an option, but even then the risk of stricture formation at the anastomotic site remains. This chapter reviews refractory benign esophageal strictures and the treatment options that are currently available.

## Introduction

Benign esophageal strictures are caused by a diversity of esophageal disorders or injuries, for example gastroesophageal reflux, radiation therapy, ablative therapy, or the ingestion of a corrosive substance. In addition, stricture formation may be a complication of esophageal resection with gastric tube formation [1, 2]. More than 80–90 % of esophageal strictures can be treated successfully with endoscopic dilation using Savary bougies or balloons. Esophageal dilation is a procedure with a very low rate of serious complications, mainly bleeding and perforation [3–5]. Unfortunately, approximately one third of patients develop recurrent dysphagia after dilation within the first year. The majority of these patients are managed with repeat dilations, depending on their complexity [2, 6].

Simple strictures are considered to be short, focal, straight, and to allow passage of a normal diameter endoscope. Examples include Schatzki rings, esophageal webs, and peptic strictures [7]. Overall, one to three dilations are sufficient to relieve dysphagia in simple strictures. Only 25–35 % of patients require additional sessions, with a maximum of five dilations in more than 95 % of patients [4]. Complex strictures are usually longer (>2 cm), angulated, irregular, or have a severely narrowed diameter. These strictures are more difficult to treat and have a tendency to be refractory or to recur despite dilation therapy. A fair number of complex strictures include circular, anastomotic strictures in the absence of endoscopic evidence of inflammation [8••, 9]. Other etiologies include radiation induced strictures, caustic strictures, and photodynamic therapy induced strictures [7].

Dysphagia is the most common symptom in patients with a benign esophageal stricture. Remarkably, most patients do not experience severe weight loss, as can be seen in malignant esophageal strictures [9]. Treatment aims to relieve symptoms, with the avoidance of complications and the prevention of recurrences. Still, dilation is the first line option to treat benign esophageal strictures. When strictures are refractory or recur, dilation therapy combined with steroid injections, incisional therapy, stent placement, self-bougienage, or surgery can be considered [10]. According to the Kochman criteria, refractory or recurrent strictures are defined as an anatomic restriction because of a cicatricial luminal compromise or fibrosis resulting in clinical symptoms of dysphagia in the absence of endoscopic evidence of inflammation. This may occur as the result of either an inability to successfully remediate the anatomic problem to a diameter of at least 14 mm over five sessions at two-week intervals (refractory); or as a result of an inability to maintain a satisfactory luminal diameter for four weeks once the target diameter of 14 mm has been achieved (recurrent). This definition is not meant to include patients with an inflammatory stricture (which will not resolve until the inflammation subsides), or those with a satisfactory diameter but having dysphagia on the basis of neuromuscular dysfunction (for example those with dysphagia due to postoperative and/or postradiation therapy) [8••].

## Treatment of benign esophageal strictures

### Dilation

The first step in managing benign esophageal strictures remains dilation with an inflatable balloon or a (Savary) bougie [4, 9, 11]. In the literature, no differences have been shown between balloon and bougie dilation in relief of dysphagia and/or recurrence of dysphagia. Also no differences have been shown in the risk of major complications [12–14]. Major complications include perforation, bleeding, and bacteremia. Perforation risk varies between 0.1 % and 0.4 % [11]. Although the majority of patients are effectively treated with up to five dilations, approximately 10 % of patients need ongoing dilations to become dilation free [8••, 15]. In order to reduce the number and burden of endoscopic dilations to become dysphagia free, various endoscopic treatment options have been suggested.

### Dilation combined with steroid injection

Adding steroid injection to endoscopic dilation into the stricture followed by dilation to avoid recurrent dysphagia has been reported to prevent stricture recurrence. This method, advocated since 1966, has shown encouraging results in patients with peptic strictures [16]. However, most of these studies were small and uncontrolled [17–19]. Randomized trials are unfortunately limited and small-sized [20–22]. Camargo, et al., randomized 14 patients with corrosive strictures allocated to steroid injection or placebo [20]. These authors did not find a difference in dilation frequency or recurrent dysphagia between the two groups. In another randomized trial, 21 patients with strictures of different etiologies were included. An increase in the dysphagia free period and periodic dilation index was reported in the steroid arm, but there was not a difference in the total number of dilations. Another study demonstrated a decrease in mean dilation frequency in patients with peptic strictures, from six dilations in the control group to two dilations in the steroid group after one year follow up [21]. Ramage, et al., performed a randomized trial comparing dilation to intralesional 4-quadrant injection of triamcinolone injections [22]. Thirty patients with peptic strictures with recurrent dysphagia after at least one dilation session were included. They concluded that dilation combined with steroid injection and gastric acid suppression therapy reduced the number of repeat dilations and the dysphagia free period, with re-dilation rates of 13 % in the steroid group versus 60 % in the control group (p = 0.01).

Hirdes, et al., recently evaluated the efficacy of intralesional triamcinolone injections combined with endoscopic dilation in a relatively large group of patients with anastomotic strictures [23•]. A total of 60 patients with untreated cervical anastomotic esophageal strictures after esophagectomy with gastric tube reconstruction and dysphagia for at least solid food were enrolled and randomized to dilation with or without steroid injections. They concluded that adding intralesional steroid injections to Savary dilation in patients with untreated benign anastomotic esophageal strictures did not result in a clinical benefit. Furthermore, an increased incidence of candida esophagitis was found in the remaining esophagus proximal to the anastomosis.

In conclusion, there is evidence that steroid injection in combination with dilation is able to reduce the risk of recurrent dysphagia in refractory benign esophageal strictures of peptic origin. Nonetheless, this result comes from various small-sized studies with poorly defined patient populations. Furthermore the optimal injection dose, technique and frequency remain to be determined. On the contrary, adding steroid to dilation was not found to be effective in anastomotic strictures. The pathogenesis of anastomotic strictures differs from that of peptic strictures, in that the former is due to ischemia whereas the latter develops as a result of inflammation and ulceration from reflux of gastric acid [24]. Steroids are suggested to locally inhibit the inflammatory response, resulting in a reduction of collagen formation [22].

### Needle knife incision

**Fig. 1:**
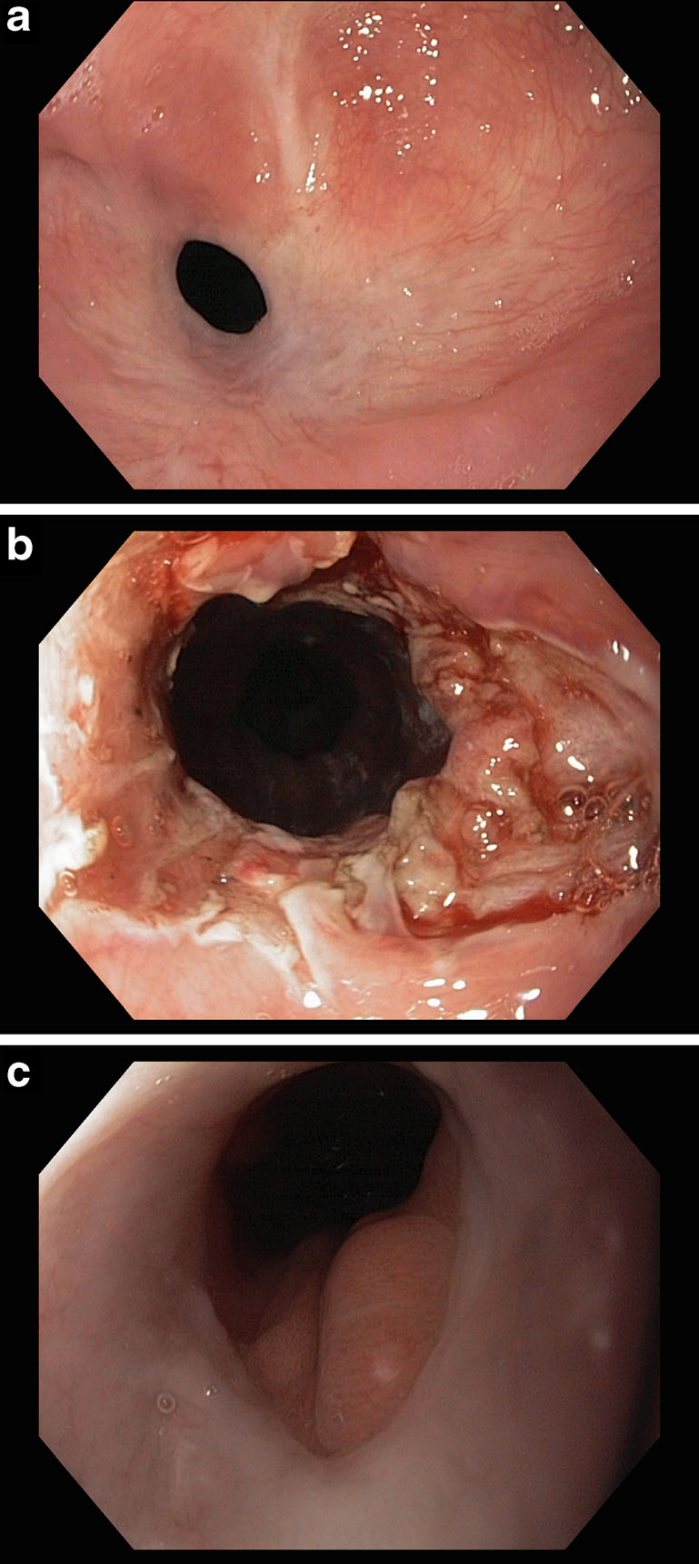
Endoscopic view of. **a** Anastomotic stricture. **b** Directly after incisional therapy. **c** After long term follow up.

Incisional therapy with a needle knife was first reported for the treatment of Schatzki rings [25, 26]. Subsequently, incisional therapy added to balloon dilation, or incisional therapy using a polypectomy snare with additional argon plasma coagulation, were shown to be effective in a small series of patients with anastomotic strictures, without the occurrence of complications [27–30]. Hordijk, et al., included 20 patients with anastomotic strictures that were refractory to dilation, and demonstrated that incisional therapy was safe and effective in simple, short strictures (<10 mm) [31]. In another study, 24 patients with anastomotic strictures without previous dilation were included. They were treated with endoscopic incisional therapy applying a transparent hood on the tip of the endoscope to enhance control and safety. After two years of follow up 87.5 % of the patients were still dysphagia free after one session [32]. In 2009, Hordijk, et al., randomized 62 patients with a primary anastomotic stricture after esophagectomy (who were not previously treated with dilation therapy), to Savary dilation or electrocautery incision. No significant difference in clinical success rates were detected between the incisional therapy and dilation therapy arms [33]. Furthermore no complications were observed after incisional therapy. So, incisional therapy can be considered as an alternative treatment in patients with a (relatively) short stenosis (Fig. 1).

### Stent placement

Dilation of an esophageal stricture with a balloon or a bougie is usually done for a period of a few seconds or some minutes. It can, however, be imagined that if the dilator can be kept in place for a longer time, the benefits of dilation may be longer lasting. In the past few years, temporary stent placement has increasingly been used for refractory benign esophageal strictures. Self-expandable plastic stents (SEPS) are FDA approved for this indication, and have been used [34, 35]. Partially and fully covered self-expandable metal stents (SEMS), although not FDA approved, are also frequently used to treat benign esophageal strictures. An alternative for SEPS and SEMS is the biodegradable stent [36], which has the advantage of not requiring removal.

#### Self-expandable metal stents (SEMS)

Uncovered SEMS were initially used for the treatment of refractory benign esophageal strictures [37–41]. In more recent years, partially or fully covered SEMS have become available and are now commonly used for this indication [35, 40, 42–44]. One of the major drawbacks of uncovered and partially covered SEMS, is that they are associated with a relatively high complication rate, mostly due to hyperplastic tissue ingrowth through the stent mesh resulting in embedding of the stent in the mucosa [45]. The complication rate of uncovered or partially stents has been reported to be as high as 80 %. The most common complications of these stents is indeed new stricture formation due to tissue ingrowth, but also stent migration, pain, gastroesophageal reflux if the stent is positioned across the gastroesophageal junction, and fistula formation [46]. Tissue ingrowth consists histologically of granulation tissue, but reactive hyperplasia and fibrous tissue are also seen [47]. Tissue reaction often results in recurrent dysphagia and may hamper stent removal. On the other hand, particularly minor tissue ingrowth may also reduce the risk of stent migration (only 12 % vs. 36 % for fully covered SEMS) [35, 48, 49•, 50, 51]. The risk of tissue ingrowth increases with stenting time, but can already be seen after one to four weeks. Tissue ingrowth can successfully be treated with the stent-in-stent method described by Hirdes, et al. Using this technique a fully covered stent is placed inside the previously placed embedded stent [49•]. The fully covered stent should have a length that at least overlaps and to have a size that is equal, or slightly larger than, the initially placed partially covered stent. Over a period of 10–14 days pressure necrosis of the hyperplastic tissue occurs as a result of friction. Hereafter, both stents can usually easily be removed.

To overcome the problem of stent ingrowth, fully covered stents (SEMS or SEPS) seem preferable for benign esophageal strictures. Currently, data on the use of fully covered SEMS is limited. In the first study performed by Eloubeidi, et al., a total of 36 stents were placed in 31 patients over a period of 16 months. A clinical success rate of 29 % was reported. A total of 47 % of these patients had no recurrence of dysphagia [48]. Bakken, et al., performed a retrospective study including seven patients with a refractory stricture. Stent migration occurred in more than half of the patients. None of the patients were treated successfully [52]. In 2011, Eloubeidi, et al., included 10 patients with a benign refractory esophageal stricture. A clinical success rate of 21 % was reported, with a migration rate of 10 % [53]. A new generation of fully covered SEMS, the fully covered Wallflex (Boston Scientific, Natick, MA), was recently evaluated by Hirdes, et al. They included 15 patients with a refractory benign esophageal stricture. The migration rate was 35 %, while tissue overgrowth was seen in 20 % of patients. Recurrent dysphagia occurred in all patients after a median of only 15 days after stent removal. These disappointing results were however most likely due to the highly refractory patient population in this study [54].

#### Self-expandable plastic stents (SEPS)

SEPS have been proposed as an alternative to SEMS to minimize hyperplastic tissue reflection. In 2010, Repici, et al., performed a pooled data analysis of all available studies on the use of SEPS for benign esophageal strictures. A total of 130 treated patients were included from 10 studies. Stent placement was technically successful in 98 % of the patients. In 52 % of patients no further dilations were required after a median follow up of 13 months after stent removal. Median stenting time in these studies was not reported. In patients with a proximal stricture the success rate was somewhat lower (33 %). As can be expected, due to the fully covered stent design, a relatively high percentage (24 %) of stents migrated within four weeks, resulting in a high rate of endoscopic re-interventions (21 %). Major complications were seen in 9 % of patients. One patient died of massive bleeding [55]. More recently, Ham, et al., published an updated systematic review. A total of 172 patients with a benign esophageal stricture were included and treated with SEPS. They found a technical success rate of 98 % and a clinical success rate of 45 % with a rate of early stent migration of 31 % [56]. It can be concluded that SEPS are effective for the treatment of refractory esophageal strictures, but the design needs further improvement to reduce the risk of migration. Moreover, the stent has a high radial and axial force, which may be the cause of an increased risk of stent-related complications to the esophageal wall, for example severe bleeding.

**Fig. 2:**
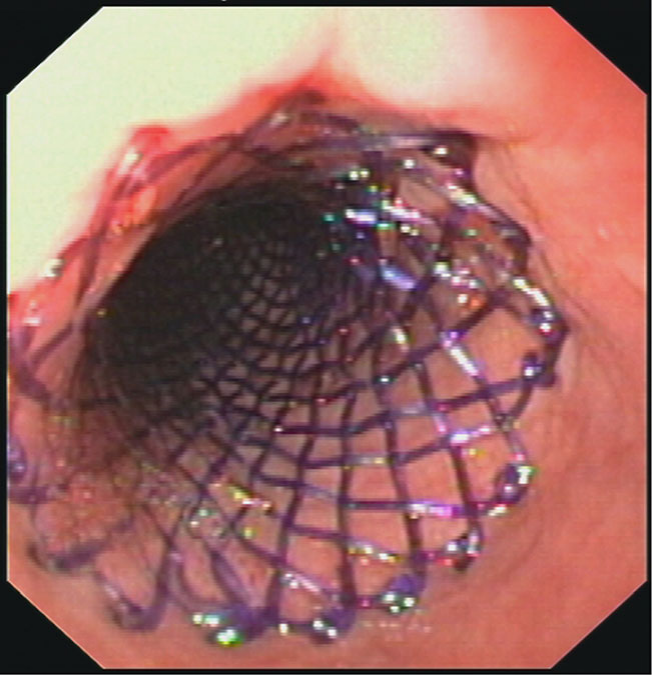
Endoscopic view of a peptic stricture with an expanding BD stent in the esophageal lumen.

In general, more studies are needed to compare different stent designs head-to-head for the treatment of benign esophageal strictures. An alternative treatment option that has recently been introduced is the placement of a biodegradable stent (Fig. 2). Van Boeckel, et al., compared biodegradable stents with SEPS, i.e., Polyflex stent (Boston Scientific, Natick, MA), in a nonrandomized head-to-head comparison. They found that both SEPSs and biodegradable stents provided long-term relief of dysphagia in 30 % and 33 %, respectively, of patients with a refractory esophageal stricture. However, biodegradable stents require fewer procedures than SEPSs [57•].

#### Biodegradable stents

Only a small number of cohort studies on the use of biodegradable stent placement in the esophagus have been published, with only a few studies including 10 or more patients [36, 57•, 58–66]. Repici, et al., included over 30 patients with a refractory benign esophageal stricture and placed an Ella BD stent (Ella CS, s.r.o., Czech Republic). Complete relief of dysphagia was reported in 43 % of patients after a median follow up of 53 weeks [36]. In this study, eight (26 %) patients had recurrent dysphagia resulting from a recurrence of the stricture. No major complications were seen. In the above-mentioned study, van Boeckel, et al., reported complete relief of dysphagia in 33 % of patients treated with a biodegradable stent after a median of 166 days. In this study, major complications occurred in four (22 %) patients (two hemorrhage and two severe retrosternal pain) [57•]. Ibrahim, et al., included 20 patients treated with an Ella BD stent. Half of them needed one or more additional procedures for recurrent dysphagia after six months of follow up [64]. Van Hooft, et al., also concluded that placement of an Ella BD stent was an effective one step treatment in 60 % (6 of 10), of patients with an anastomotic stricture in the esophagus. No major complications were reported [66]. The other 40 % of the patients required endoscopic dilation because of stricture related recurrent dysphagia. Recently, Hirdes, et al., reported the efficacy and safety of sequential Ella BD stent placement in 28 patients with a refractory benign strictures [58]. A total of 59 biodegradable stents were placed in these patients. After initial stent placement patients remained dysphagia free for a period of 90 days, while after six months still 25 % of patients were dysphagia free. After placement of a second biodegradable stent in patients with recurrent stricture formation, patients remained dysphagia free for a median period of 55 days. After six months only 15 % of these patients were still dysphagia free. After a third biodegradable stent placement, the median dysphagia free period was 106 days but none of the patients remained dysphagia free after a period of six months. Major complications occurred in 29 %, 8 %, and 28 % of patients after one, two, and three Ella BD stents respectively. From these studies it can be concluded that a single biodegradable stent is only temporarily effective in the majority of patients. The relatively low radial force and degradable nature of these stents may contribute to early stricture recurrence [67]. Stent placement was also found to be associated with considerable complications, like retrosternal pain and vomiting. However, in selected patients with a refractory benign esophageal stricture, sequential biodegradable stent placement can be an effective alternative to avoid the burden of frequent serial dilations. Further (prospective), studies including larger numbers of patients, and comparing biodegradable stents with fully covered SEMS (or SEPS), are needed. In those studies patient satisfaction and costs should be evaluated besides efficacy and safety.

#### Optimal duration of stent placement in refractory benign esophageal strictures

The optimal duration of stent placement for treating refractory benign esophageal strictures is unknown, but likely depends on a number of variables, such as stricture type, severity of the inflammation, stricture length, and stent type. These factors should be evaluated in all patients. The general principle is to leave the stent in place until the inflammation is resolved. In strictures longer than 5 cm or those due to ischemic injury, dilation for a period of at least 8–16 weeks is recommended. For shorter strictures and other etiologies shorter stenting times can be recommended, but still these strictures may also be refractory. Only fully covered stent designs can safely be removed after a prolonged time of stenting. When partially covered stents are used, repeat endoscopy should be performed at 2–4 week intervals to evaluate embedding of the stent in the wall. After biodegradable stent placement, a completely different treatment strategy can be followed. Only when patients treated with a biodegradable stent present with recurrent dysphagia should a repeat endoscopy be performed. In most cases this means that the stent is dissolved and a new stent, either biodegradable or SEMS, can be placed.

## Treatment selection (algorithm)

**Fig. 3:**
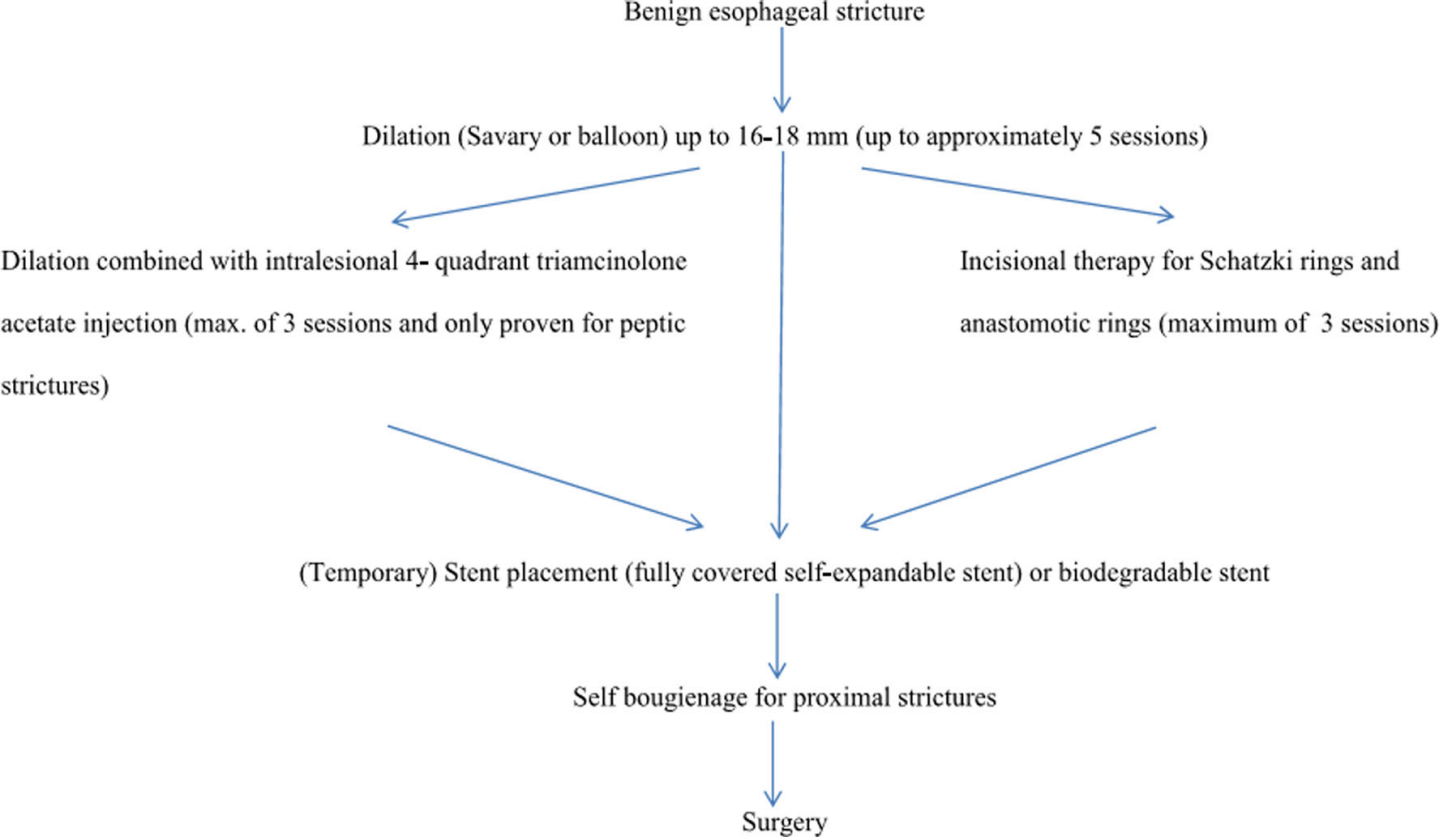
Recommended treatment scheme for patients with benign esophageal stricture.

In absence of evidence based treatment guidelines for patients with dysphagia due to refractory benign esophageal strictures [68, 69], an algorithm has been suggested for the therapeutic management of patients with benign dysphagia [10, 70], which is shown in Figure 3. Dilation remains the first choice as the least invasive approach with a low complication rate (0.001–0.040 %) [69]. If the selected approach is not sufficient a next step in the algorithm should be discussed with the patient, i.e., dilation with steroids, incisional therapy for selected strictures, or stent placement. If still refractory, self-bougienage can be proposed to patients with a stenosis in the proximal esophagus [71, 72]. An ultimate step in the management of (refractory), benign esophageal strictures includes surgery, taking into account that even after a surgical solution the risk of stricture formation remains [24–26]. In our experience, the majority of benign strictures can be managed by non-surgical means.

Randomized trials are needed to determine the optimal treatment strategy in patients with refractory and recurrent benign esophageal strictures. One such trial includes a comparison between Savary or balloon dilation therapy and stent placement, either a fully covered SEMS or biodegradable stent, to determine whether stent placement could be positioned at an earlier stage in the treatment algorithm. Furthermore, biodegradable stents should be compared with fully covered stent designs (as discussed earlier). Finally, the use of a (locally applied) treatment aiming to improve oxygenation (anastomotic strictures), and/or to reduce the inflammatory process in strictures, could be an important step.

## Conclusion

The treatment of refractory benign esophageal strictures remains a challenge for clinicians. Dilation of the stricture with Savary or balloon remains the first step. Dilation combined with intra lesional injections with steroids can be considered for peptic stenosis, while incisional therapy is found to be effective for Schatzki rings and anastomotic strictures. After failure of these therapeutic options stent placement can be considered. A final step includes self bougienage or surgery. Following this treatment algorithm means that most patients with a difficult to treat esophageal stricture can be managed without an invasive surgical procedure.
